# Valorization of Chestnut By-Products: Extraction, Bioactivity, and Applications of Shells, Spiny Burs, and Leaves

**DOI:** 10.3390/life16010140

**Published:** 2026-01-15

**Authors:** Stefania Lamponi, Roberta Barletta, Annalisa Santucci

**Affiliations:** 1 Department of Biotechnology, Chemistry & Pharmacy, University of Siena, Via Aldo Moro 2, 53100 Siena, Italy; 2 SienabioACTIVE, University of Siena, Via Aldo Moro 2, 53100 Siena, Italy; 3 ARTES 4.0, Viale Rinaldo Piaggio 34, 56025 Pontedera, Italy

**Keywords:** *Castanea sativa*, chestnut by-products, spiny burs, polyphenols, circular bioeconomy

## Abstract

The European chestnut (*Castanea sativa* Mill.) industry generates substantial amounts of underutilized biomass, including shells, leaves, and spiny burs. Distinguishing itself from existing literature, this review presents a novel, integrated life-science analysis that redefines these by-products as a complementary ‘bioactive triad’, ranging from metabolic regulators to anti-virulence agents, rather than interchangeable sources of polyphenols. Although traditionally discarded, these by-products are rich sources of polyphenols, ellagitannins, and flavonoids, with promising potential for nutraceutical, cosmetic, and pharmaceutical applications. This review examines recent advances in the valorization of chestnut by-products, focusing on extraction strategies, chemical profiles, and biological activities. Shell valorization has increasingly shifted toward green extraction technologies, such as subcritical water extraction and deep eutectic solvents, which strongly influence bioactive recovery and composition. Chestnut leaves emerge as a sustainable resource enriched in hydrolysable tannins with anti-inflammatory and quorum sensing-inhibitory properties, particularly relevant for dermatological applications. Spiny burs, often the most phenolic-rich fraction, display marked antioxidant activity and the ability to potentiate conventional antibiotics against pathogens such as *Helicobacter pylori*. Despite these promising features, major challenges remain, including cultivar-dependent chemical variability, the predominance of in vitro evidence, and safety concerns related to the accumulation of potentially toxic elements. Overall, while chestnut by-products represent valuable resources within circular bioeconomy frameworks, their successful industrial and practical translation will require standardized extraction protocols, robust bioavailability assessments, and well-designed in vivo and clinical studies to ensure safety and efficacy.

## 1. Introduction

*C. sativa* Mill., commonly known as the European chestnut, is a keystone agroforestry species with long-standing nutritional, ecological, and socio-economic relevance in Southern Europe [1]. Global chestnut production exceeds 2.3 million tons per year, with Europe contributing approximately 12% of the total (over 290,000 tons), particularly through Italian and Portuguese cultivation systems [2,3]. Chestnut fruits are widely appreciated as seasonal food products in Mediterranean countries and are traditionally valued for their nutritional and cultural importance [4].

Alongside nut production, chestnut cultivation and industrial processing generate substantial quantities of residual biomass, including shells, spiny burs, and leaves, which together account for a significant fraction of the harvested material [5]. Historically, these by-products have been underutilized or discarded, often relegated to low-value applications such as combustion or mulching, despite their chemical richness [6]. In the last 10 years, however, the transition toward circular bioeconomy models has promoted a re-evaluation of agro-industrial residues as renewable resources for high-value applications, emphasizing waste reduction, resource efficiency, and sustainability [7].

Within this framework, chestnut by-products have attracted increasing scientific interest as sources of bioactive compounds, particularly polyphenols such as hydrolysable tannins, ellagitannins, and flavonoids [8]. These compounds are widely associated with antioxidant, anti-inflammatory, antimicrobial, and metabolic regulatory activities, supporting their potential use in food, nutraceutical, cosmetic, pharmaceutical, and veterinary applications [9]. Comprehensive analyses of chestnut residues have highlighted their significant phytochemical diversity and biological potential, reinforcing their relevance within sustainable bio-based value chains [10].

Importantly, chestnut by-products are not chemically or biologically equivalent. Shells, spiny burs, and leaves exhibit distinct phytochemical profiles that translate into different biological activities and application potentials [11]. Spiny burs are generally reported as the most phenolic-rich fraction, characterized by high concentrations of ellagitannins such as castalagin and vescalagin, which underpin their strong antioxidant and antimicrobial properties [10]. Leaves, which can be harvested annually without compromising tree vitality, represent a particularly sustainable resource and are rich in hydrolysable tannins and flavonoid glycosides, supporting both traditional uses and emerging health-related applications [12]. Shells, although often less phenolic than spiny burs, remain relevant matrices due to their abundance and documented bioactive potential [9].

In parallel with chemical characterization, increasing emphasis has been placed on extraction strategies that align with sustainability goals. Green extraction technologies, including ultrasound- and microwave-assisted extraction and other low-impact approaches, have been explored to enhance the recovery of phenolic compounds while reducing solvent consumption and environmental burden [3]. However, extraction conditions strongly influence both yield and composition, contributing to variability in reported bioactivities and complicating direct comparison between studies [5,10].

Despite the growing body of literature, critical gaps remain that limit industrial translation. Most recent studies focus on single-matrix characterization without standardized extraction protocols, leading to data that is difficult to compare [5]. Furthermore, while the antioxidant potential is well-documented in vitro, there is a scarcity of in vivo and clinical evidence validating these effects in complex biological systems [11,12,13]. Additionally, the comparative bioactivity of shells, spiny burs, and leaves extracted under identical conditions remains largely unexplored. Addressing these gaps is essential for translating promising laboratory findings into reliable, safe, and effective applications.

While previous reviews have primarily focused on individual chestnut by-products or on technological and food-related aspects, an integrated biological comparison of shells, spiny burs, and leaves is still lacking. The novelty of the present review lies in its life-science-oriented perspective, which systematically examines these matrices as a complementary bioactive triad, linking extraction strategies and phytochemical profiles to distinct biological mechanisms and application pathways. Rather than treating chestnut residues as interchangeable sources of polyphenols, this review emphasizes their functional specialization and translational relevance, particularly in relation to emerging challenges such as antimicrobial resistance, chronic inflammation, and metabolic disorders [10,11].

The section dedicated to chestnut leaves is intentionally more concise than those on shells and spiny burs. This reflects the current state of the literature, which prioritizes specific biological mechanisms, such as anti-virulence and dermatological effects, over broad extraction surveys. By providing a focused, mechanism-driven overview, this review aims to highlight the strategic importance of leaves without redundancy, while identifying clear priorities for future targeted research [12,13].

## 2. Chestnut Shells

**Figure 1:**
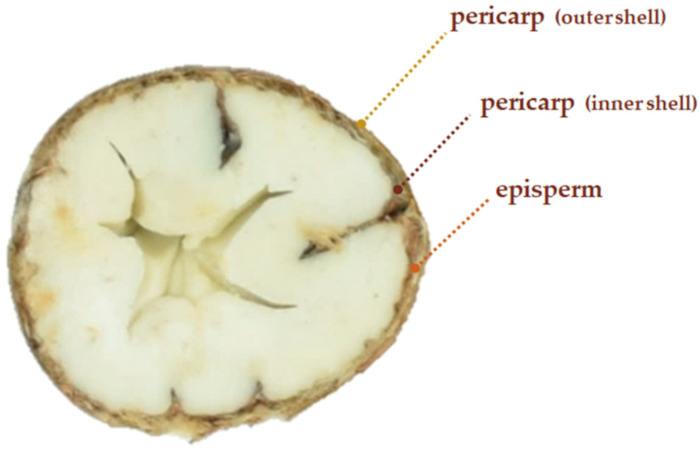
Cross-sectional view of the chestnut (*C. sativa* Mill.) fruit, highlighting the anatomical layers that give rise to shell by-products, including the outer pericarp, inner pericarp, and episperm. Adapted from Ferrara et al. (2022) [16].

Chestnut shells (comprising the outer pericarp and inner integument, Figure 1) represent a significant waste stream generated primarily during the industrial peeling process [4].

Chemically, shells are characterized by a distinct polyphenolic profile dominated by phenolic acids, such as gallic and ellagic acid, and condensed tannins, differing from the hydrolysable tannin-rich profile of the spiny burs [14,15].

### 2.1. Extraction Methods

Scientific investigations have explored a wide range of methodologies to maximize the recovery of bioactives from chestnut shells, spanning conventional solvent-based extractions and advanced green technologies [10]. Classical extractions using ethanol or hydroethanolic mixtures remain widely employed as benchmarks. In the Verdole cultivar of *C. sativa*, fractionation of the shell into outer shell, inner shell, and episperm demonstrated that the outer pericarp contains the highest levels of total phenolics, flavonoids, and condensed tannins, yielding extracts with strong DPPH, ABTS, and iron-reducing antioxidant capacity [16]. Ultrasound-assisted maceration further improves extraction efficiency, confirming the importance of anatomical fraction, particle size, and pre-treatment. However, conventional maceration is characterized by high solvent consumption, long extraction times, and limited selectivity. Moreover, the heterogeneity of maceration conditions reported in the literature complicates direct comparison between studies and hampers process scale-up [10].

Among green technologies, subcritical water extraction (SWE) has emerged as one of the most extensively investigated approaches for chestnut shell valorization. Under subcritical conditions, water exhibits enhanced solubilizing power for less polar phenolics and improved diffusion through the lignocellulosic matrix. Two main optimization strategies have been reported. Moderate conditions (temperatures ranged between 110 and 140 °C) yield extracts rich in gallic acid, catechin, epicatechin, protocatechuic acid, and rutin, with high antioxidant activity and limited tannin degradation [17]. In contrast, response surface methodology identified harsher conditions (220 °C for 30 min) as optimal for maximizing total phenolic content (315–497 mg GAE/g DW) and antioxidant parameters [18]. Under these conditions, the phenolic profile is dominated by low-molecular-weight compounds such as pyrogallol and protocatechuic acid, indicating partial depolymerization of ellagitannins. These contrasting “optimal” conditions reflect different priorities, preservation of native tannins versus maximization of global antioxidant readouts, and result in extracts that are bioactive but chemically and functionally non-equivalent [17,18]. SWE extracts have also been applied to functional food enrichment, such as cookies, where phenolics are retained with preserved antioxidant activity and acceptable sensory properties [19]. Under subcritical conditions, water exhibits enhanced solubilizing power for less polar phenolics due to a decrease in the dielectric constant, while simultaneously disrupting the lignocellulosic matrix to improve mass transfer and bioactive release.

Supercritical fluid extraction (SFE), mainly using supercritical CO_2_ with co-solvents, has been explored as an alternative green technology capable of selectively extracting less polar compounds while also recovering phenolic fractions. An SFE-derived chestnut shell extract retained antioxidant, antiradical, hypoglycemic, and neuroprotective activities after in vitro gastrointestinal digestion, despite a reduction in total phenolic content corresponding to approximately 30% bioaccessibility [20]. LC–Orbitrap MS revealed extensive biotransformation of complex phenolics into hydroxybenzoic, phenylpropanoic, and phenylacetic acids during digestion [20]. In intestinal permeability models, SFE extracts showed hypoglycemic, hypolipidemic, and neuroprotective effects, with untargeted metabolomics indicating the permeation of phenolic acids, flavonoids, and coumarins alongside lipid components [21]. These findings support SFE extracts as promising nutraceutical candidates, while also highlighting their distinct phenolic fingerprint compared to SWE and conventional extracts.

More recently, deep eutectic solvents (DES), particularly natural DES, have been applied to the extraction of chestnut shell pigments. In *C. mollissima* shells, ultrasound-assisted extraction using choline chloride–lactic acid produced pigment-rich extracts with good color stability, notable total phenolic content, and strong antioxidant activity [22]. HPLC–MS analysis of the shell matrix identified a diverse range of compounds, including flavonoids, procyanidins, ellagic acid derivatives, and coumarins, with total phenolic contents confirmed by quantitative profiling [22]. DES-based extracts displayed superior pigment stability compared to ethanol or other DES systems, expanding shell valorization toward natural colorant applications, although compositional differences from conventional phenolic extracts must be considered when evaluating bioactivity.

Ultrasound-assisted extraction (UAE) and microwave-assisted extraction (MAE) are recognized as rapid and energy-efficient techniques. UAE with water or hydroethanol generally yields phenolic-rich shell extracts by exploiting acoustic cavitation, which disrupts cell walls and enhances solvent penetration. For instance, MAE with water produces extracts with significantly higher reducing power compared to conventional maceration, as rapid internal heating accelerates the release of intracellular antioxidants [3].

### 2.2. Chemical Composition of Chestnut Shell Extracts

Across *C. sativa* and *C. crenata*, chestnut shells are characterized by a dense and complex polyphenolic profile, complemented by lipids, vitamin E, amino acids, and non-digestible carbohydrates [10,23]. Phenolic acids are central components; gallic acid is consistently the dominant hydroxybenzoic acid, accompanied by protocatechuic, syringic, and vanillic acids, and by hydroxycinnamic derivatives such as caffeic, chlorogenic, and ferulic acid [16,18]. Flavonoids include catechin and epicatechin as major flavanols, and quercetin, rutin, kaempferol, and isorhamnetin glycosides as key flavonols [16]. Ellagitannins form the hallmark of chestnut shell chemistry. Vescalagin, castalagin, chestanin, galloyl-cretanin, and related structures, together with ellagic acid and its glycosides, define the ellagitannin signature of *Castanea*by-products [10,14,15]. Under harsher SWE conditions, these high-molecular-weight tannins are partially depolymerized into pyrogallol and methyl gallate, which contributes to antioxidant capacity but alters the metabolic and sensory profile [18].

Beyond phenolics, chestnut shells contain vitamin E, essential amino acids (notably arginine and leucine), lignin, and substantial amounts of structural carbohydrates (cellulose, hemicellulose) and oligosaccharides, which may act as prebiotic dietary fiber [9,10]. The combination of polyphenols and non-digestible carbohydrates may underline the prebiotic potential suggested in some studies, but this has not yet been comprehensively validated in human models. Metabolomic profiling has highlighted substantial intra-species and inter-species variability. A UPLC-QTOF-MS study of different *C. crenata* cultivars (Okkwang, Porotan, Ishizuuchi, etc.) showed that some cultivars have particularly high levels of ellagic acid derivatives, ellagitannins, flavonoids, and gallic-acid derivatives, with antioxidant capacity correlating with these metabolites [23]. The same work revealed that whole shells (outer + inner) can contain higher levels of certain phenolic acids and flavonoid glucosides than inner shell alone, and that whole shell extracts more effectively reduced intracellular ROS in cell models [23]. This reinforces the need to carefully define which shell fraction is used in each study. A recent comprehensive review has also emphasized that *C. sativa* shells contain 2.7–5.2% polyphenols (*w*/*w*) and roughly 36% sugars, and that they are excellent sources of both condensed and hydrolysable tannins, as well as phenolic acids and flavonoids [10].

### 2.3. Biological Activity

Chestnut shell extracts have consistently shown high antioxidant activity in chemical assays (DPPH, ABTS, FRAP, ORAC), often surpassing many fruit and vegetable extracts when expressed per gram of dry extract. RSM-optimized SWE extracts reached exceptionally high values for reducing power and radical scavenging, alongside strong superoxide anion scavenging activity [18]. These extracts demonstrated potent scavenging capabilities against reactive species with no detectable intestinal cytotoxicity at effective concentrations [18]. In food applications, cookies enriched with SWE chestnut shell extract displayed significantly higher antioxidant capacity than control cookies, with activity maintained after baking and simulated digestion [19]. Pigment-rich DES extracts from *C. mollissima* also show strong antioxidant activity and good stability, suggesting potential for simultaneous colorant and antioxidant functions [22].

In human intestinal epithelial cells, *C. sativa* by-product extracts significantly reduce the release of pro-inflammatory mediators, inhibit NF-κB activation, and promote responses that support barrier function [24]. In oral epithelial models, chestnut shell SWE extracts display cytoprotective and antioxidant effects that support their proposed use in preventing or alleviating oral mucositis [17]. In vivo studies in rats have shown that oral administration of phenolic-rich shell extracts improves antioxidant status in liver, kidney, and serum, increases antioxidant enzyme activity, and reduces lipid peroxidation [25]. Furthermore, complementary work using *C. crenata* inner shell extracts in mouse models of allergic asthma and emphysema has shown suppression of Th2 cytokines and attenuation of tissue inflammation [26,27]. Chestnut shell extracts also display broad but moderate antimicrobial activity against several oral and opportunistic pathogens, including *Staphylococcus aureus* and *Escherichia coli* [17]. Extracts from chestnut by-products also inhibit foodborne bacteria such as *Pseudomonas aeruginosa* and *Enterobacter cloacae* [3]. The antimicrobial effect has been attributed to multiple mechanisms: membrane disruption, enzyme inhibition, metal ion chelation, and interference with cell-wall integrity, driven primarily by galloylated tannins, ellagic acid derivatives, catechins, and other polyphenols. However, critical evaluation shows that the MIC values are relatively high compared with conventional antibiotics or some essential oils, which limits the feasibility of using chestnut shell extracts as stand-alone antimicrobial agents. In food systems, where higher concentrations are acceptable and antioxidant and antimicrobial effects can act synergistically, these extracts appear more promising as natural preservative co-factors rather than single-agent antimicrobials.

### 2.4. Digestion, Bioaccessibility, and Safety

A major gap in earlier work has been the translation from in vitro assays to real in vivo bioactivity. However, it has been observed that anti-inflammatory effects are maintained, at least in part, after simulated gastrointestinal digestion [28]. Simulated gastrointestinal digestion of a SWE chestnut shell extract showed that phenolic concentrations actually increased from the oral to gastric and intestinal phases, likely due to release from the matrix, with overall bioaccessibility reaching about 40% [28]. Ellagic acid remained the main phenolic, while a pyrogallol–protocatechuic acid derivative appeared only after digestion, indicating digestion-driven formation of new metabolites. Intestinal permeation studies indicated that about 23% of ellagic acid crossed the epithelial monolayer after 240 min [28]. The digested extract maintained mild hypoglycemic and substantial neuroprotective effects, and up-regulated antioxidant enzymes while reducing lipid peroxidation in cell models.

Regarding safety, *C. sativa* has been proposed as a biomonitor of potentially toxic elements (PTEs) such as As, Hg, Tl, and Pb in mining-impacted areas. A 2024 study in the Alpi Apuane mining district (Italy) showed that chestnut trees can tolerate very high soil PTE levels and that these elements are distributed differentially in leaves, bark, wood, nuts, and shells. Tl and Hg were detected in all tissues, As mainly in leaves, wood, and nuts, and Pb primarily in bark [29]. This implies that shells harvested from contaminated areas may co-extract metals and metalloids together with polyphenols, and that stringent sourcing and metal screening are essential if shell extracts are to be used in food or nutraceutical products. Furthermore, the high phenolic density of shell extracts may lead to strong protein binding and interaction with digestive enzymes, nutrients, and drugs.

### 2.5. Potential Biotechnological Applications of Chestnut Shells

Chestnut shells have emerged as a versatile matrix for multiple biotechnological applications, transitioning from simple biomass waste to high-value functional ingredients (Table 1). In the food industry, they serve as a sustainable source of nutraceuticals and functional additives; for instance, Subcritical Water Extraction (SWE) extracts rich in gallic acid, ellagic acid, and catechins have been successfully incorporated into cookies, retaining significant antioxidant capacity and sensory acceptability after baking [19]. Beyond their antioxidant potential, shells contain non-digestible oligosaccharides that suggest a promising role as prebiotic dietary fibers, potentially modulating gut health [9]. Innovations in green chemistry have further expanded their utility; Deep Eutectic Solvents (DES) have been employed to recover stable pigments from shells, validating their use as natural colorants for textile, cosmetic, and food dye applications [22]. Furthermore, their robust profile of hydrolysable tannins and demonstrated in vivo metabolic effects, such as hypoglycemic and hypolipidemic activities, position them as viable candidates for the development of anti-aging supplements and metabolic regulators [25,30]. However, the implementation of these applications requires rigorous safety protocols, as shells can bioaccumulate potentially toxic elements from soil, necessitating strict heavy metal screening prior to formulation [29].

## 3. Chestnut Spiny Burs

### 3.1. Sampling and Extraction Methodologies

The efficient valorization of *C. sativa* spiny burs necessitates a deep understanding of their metabolite profile and the extraction methods required to efficiently isolate bioactive compounds. Various techniques were employed, ranging from classical maceration to innovative, high-efficiency, and green methods. Spiny bur samples were collected across diverse geographical and genetic origins to account for natural variability. These include the spiny burs of *C. sativa* Mill. collected in 2022 from a chestnut orchard of the Longal variety, located in the Northeast of Portugal, at Vinhais municipality [3]; the Italian ‘*Marrone di Roccadaspide*’ PGI cultivar from Salerno, Italy [11]; mixed cultivars from the experimental grove of Granaglione, Bologna [31]; samples from the *Campo dei Fiori* area (Varese, Italy) [24]; and PGI samples from the Monte Amiata region (Tuscany, Italy) [32]. Regardless of origin, the material is typically cleaned, air-dried at room temperature, milled to a fine powder, and stored at low temperatures (−20 °C to −80 °C) to preserve bioactive integrity.to preserve bioactive integrity.

Literature showcases a variety of extraction strategies. One comprehensive study by Cerulli et al. (2021) [10] applied sequential extraction, where dried spiny burs were extracted at 25 °C using solvents of increasing polarity using solvents of increasing polarity, petroleum ether, chloroform, and finally, methanol (MeOH), yielding a crude extract used for detailed phytochemical profiling [11].

Traditional Maceration (MAC) remains a standard approach where hydro-alcoholic extracts were prepared from the Portuguese spiny burs at 40 °C utilizing 80% ethanol and 20% water (80:20, *v*/*v*) [3]. Comparison with innovative methods showed that Microwave-Assisted Extraction (MAE) at 80 °C significantly reduced extraction time while maintaining comparable phenolic yields [3].

An alternative common approach involved a MeOH/H_2_O (1:1) mixture, followed by sonication and centrifugation [31]. To enhance sustainability and efficiency, innovative methods were compared using the ‘Longal’ cultivar from Rodrigues et al. (2023) [3]. These included Ultrasound-Assisted Extraction (UAE), performed with 80% ethanol (UAE-HE) or ultrapure water (UAE-W) utilizing a 25 kHz ultrasonic system, and Microwave-Assisted Extraction (MAE), conducted using ultrapure water (MAE-W) in a closed vessel at 80 °C [3]. Recently, to ensure reproducibility and maximize bioactive recovery while adhering to green chemistry principles, Frusciante et al. (2024) developed an eco-friendly ultrasound protocol for spiny burs from the Monte Amiata region to obtain an aqueous extract [32]. This optimized methodology involved suspending the powdered spiny burs in water and subjecting the mixture to 20 kHz ultrasonic waves for 3 h at room temperature. The resulting aqueous extract was subsequently freeze-dried to obtain a stable powder. This water-based UAE method offers significant advantages over traditional maceration with organic solvents, including reduced environmental impact, non-toxicity, lower maintenance costs, and scalability for industrial applications [32]. Furthermore, spiny burs from the Campo dei Fiori area were processed using Cryogenic Grinding followed by sonication in a 50:50 ethanol/water solution, with a good extraction yield [24]. Regardless of the technique, extracts were typically separated from the solid residues, concentrated (often by vacuum evaporation of ethanol), and finally subjected to freeze-drying to obtain stable, dry extracts.

### 3.2. Chemical Composition of Spiny Bur Extracts

The chemical composition of *C. sativa* spiny bur extracts is consistently reported as highly complex and rich in polyphenols across multiple studies. Spectrophotometric and colorimetric analyses demonstrate that spiny burs are among the most phenolic-rich chestnut by-products. For instance, methanolic spiny bur extracts analyzed via Folin–Ciocalteu assay showed a TPC of 580.44 mg GAE/g, with a total tannin content (TT) of 276.44 mg GAE/g and a total flavonoid content (TFC) of 87.19 mg rutin/g [11]. Similarly, the total polyphenol index (TPI) determined in hydroalcoholic spiny bur extracts ranged from 117.5 mg/g dry weight up to 580.44 mg GAE/g [11,24], highlighting considerable variability depending on the extraction method. Other studies of chestnut by-products reported comparable values for related matrices, such as shells and bark, further confirming that spiny burs are a major reservoir of phenolic compounds [12]. In addition, aqueous ultrasound-assisted extraction of PGI *C. sativa* spiny burs from Monte Amiata yielded a TPC of 243.98 ± 17.77 mg GAE/g and a TFC of 27.54 ± 0.60 mg QE/g, with notable antioxidant activity, exhibiting a reducing power of 272.12 mg AAE/g and radical scavenging IC50 values of 8.16 µg/mL (ABTS) and 29.57 µg/mL (DPPH), significantly lower than the standard Trolox controls [32]. These values indicate that extraction method and solvent choice significantly influence the polyphenolic yield and antioxidant performance of spiny burs.

Characterization of the specific compounds responsible for these high phenolic values has been performed using complementary chromatographic and spectrometric techniques. In methanolic spiny burs, high-resolution LC-ESI/LTQ-Orbitrap/MS/MS and LC-ESI/QTrap/MS/MS revealed five main classes of specialized metabolites, dominated by hydrolysable tannins, including galloyl-glucose derivatives (digalloyl- and trigalloyl-glucose isomers) and ellagitannins (ETs) such as castalagin, vescalagin, stachyurin, castacrenin B, trigalloyl glucose isomer, chesnatin, chestanin, cretanin, and galloyl cretanin [11,24]. Smaller contributions came from flavonoids, including glycosylated derivatives of quercetin, isorhamnetin, and kaempferol, as well as other phenolics such as ellagic acid and its glucosides, and triterpenoids like bartogenic acid, highlighting the structural diversity of spiny burs. Unique to spiny burs, polar lipids (including phospholipids, glycolipids, and sphingolipids) were also detected, underscoring their potential as a source of bioactive lipids. Further studies using HPLC-DAD-(ESI)MS/MS and UPLC-MS/MS [3,32] confirmed spiny burs as the richest chestnut by-product in terms of phenolic complexity and abundance, with hydrolysable tannins forming the dominant fraction, accompanied by smaller amounts of condensed tannins and flavonoids. Key ellagitannins included bis-HHDP-glucose and its structural isomers, along with galloyl-bis-HHDP-glucose, digalloyl-HHDP-glucose, and other multi-acylated glucose derivatives, all distinguishable through specific fragmentation pathways. Ellagic acid derivatives, both methylated and glycosylated, were also prevalent, alongside gallic acid and several gallotannins, further enriching the polyphenolic matrix. The flavonoid fraction, including quercetin-3-*O*-rutinoside and quercetin-3-*O*-glucoside, complemented the tannin-rich profile, though it did not dominate as in other chestnut tissues. Comparing these studies, several trends emerge: spiny burs consistently exhibit higher phenolic content and antioxidant activity than other chestnut by-products, including shells and leaves. While absolute values vary with extraction solvent and method, ranging from aqueous ultrasound-assisted extraction [32] to methanol-based methods [11], the chemical fingerprint remains dominated by ellagitannins, ellagic acid derivatives, and gallotannins, with flavonoids and triterpenoids present in smaller but significant amounts.

### 3.3. Biological Activity

The biological activities of *C. sativa* spiny bur extracts have been widely investigated across diverse in vitro models, consistently revealing antioxidant, anti-inflammatory, and antimicrobial properties closely linked to their polyphenolic composition [10,11,31]. Methanolic extracts of spiny burs demonstrated strong antioxidant capacity in spectrophotometric assays, including DPPH, TEAC, and FRAP, with intracellular ROS scavenging confirmed in immune cells [10]. Notably, spiny bur extracts significantly reduced ROS levels, outperforming reference standards like quercetin at non-cytotoxic concentrations [10]. This potent activity is attributed to the high number of hydroxyl groups in castalagin and vescalagin, which facilitate electron donation and radical stabilization, thereby neutralizing oxidative stress more effectively than simple phenols.

Anti-inflammatory effects were demonstrated through modulation of NF-κB signaling and nitric oxide (NO) production [10,31]. Extracts reduced LPS-induced NF-κB activation to levels comparable to the corticosteroid prednisone and suppressed NO production in macrophages without cytotoxicity [10].

Complementing these findings, aqueous ultrasound-assisted extracts, representing a sustainable green extraction approach, have demonstrated high biocompatibility in fibroblast and macrophage models while exerting potent antioxidant activity [32]. These aqueous extracts induced a dose-dependent suppression of oxidative stress and inflammatory mediators, mechanically driven by the downregulation of iNOS expression and the inhibition of NF-κB p65 nuclear translocation [32]. Furthermore, computational in silico analyses supported these biological observations by identifying ellagic acid as a high-affinity ligand for specific kinases involved in inflammatory signaling pathways [32].

Further mechanistic insights in microglial cells showed that spiny bur extracts downregulated surface TLR4 expression (≈54–58%) and reduced TLR4 and CD14 mRNA levels [31]. This attenuation of TLR4 signaling inhibited NF-κB phosphorylation (reducing activation to ≈62%) and decreased transcription of downstream inflammatory mediators [31]. TLR4 expression and reduced transcription of downstream inflammatory mediators, resulting in decreased production of inflammatory prostaglandins [31]. Additional antioxidant evaluation confirmed the high efficiency of spiny bur extracts, particularly in preventing lipid peroxidation, where they surpassed other chestnut fractions [3]. Hydroethanolic extracts exhibited elevated phenolic levels and were shown to be non-hepatotoxic [3]. Antimicrobial activity was also observed for microwave-assisted extracts, which displayed bactericidal effects against *E. cloacae*, *P. aeruginosa*, and *S. aureus*, and exerted bacteriostatic effects against *Listeria monocytogenes* [3]. Additionally, hydroalcoholic extracts displayed bactericidal activity against *H. pylori*, synergized with clarithromycin, and showed additive effects with metronidazole [33]. Mechanistic studies demonstrated that spiny bur extracts compromise bacterial membrane integrity and enhance antibiotic uptake, underscoring their capacity to potentiate antibiotic efficacy against resistant strains [33,34].

### 3.4. Potential Biotechnological Applications

**Figure 2:**
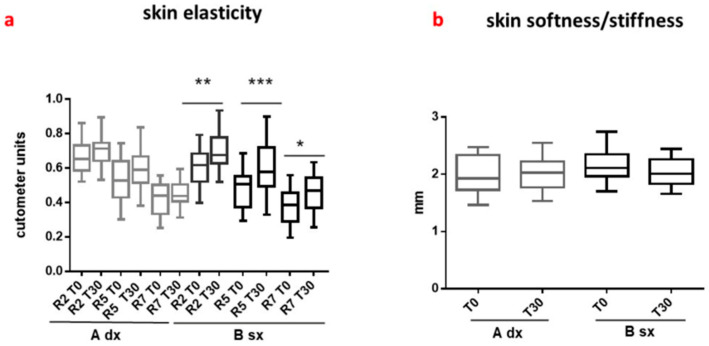
In vivo dermatological efficacy of *C. sativa* spiny bur extract. The graphs illustrate the improvement in (**a**) skin hydration and (**b**) skin elasticity in human volunteers treated with a topical emulsion containing spiny bur extract compared to a placebo over 30 days. Statistical significance was determined using ANOVA followed by Bonferroni post hoc test (*p* < 0.05, ** *p* < 0.01). Error bars indicate standard error of the mean (SEM). Asterisks indicate statistically significant differences compared to the control (* *p* < 0.05, *** *p* < 0.001). The data confirms the potential of spiny bur-derived tannins as anti-aging functional ingredients. Adapted from Esposito et al. (2021) [35].

The abundance of phenolic acids, flavonoids, and tannins in chestnut spiny burs, strongly associated with antioxidant and anti-inflammatory effects, positions them as valuable, sustainable ingredients for the cosmetic and dermatological sectors [8,35]. The skin is particularly vulnerable to oxidative stress, making the incorporation of natural antioxidants highly desirable for preventing photo-damage and inflammatory disorders [8,35]. To effectively harness these bioactives, extracts have been successfully incorporated into versatile delivery systems, specifically hydrogels and oil-in-water (O/W) emulsions. Hydrogel-Based Formulations containing moderate extract concentrations (25–50%) exhibited pH values compatible with skin application, favorable rheological, textural, and moisture characteristics, and retained high total phenolic and flavonoid content, which correlated strongly with in vitro antioxidant activity. Microbiological analysis confirmed stability, meeting ISO 17516:2014 standards [8,36]. Oil-in-Water Emulsion Formulations involved a stable hydroalcoholic extract (CSE), incorporated at 0.3% *w*/*w*. The resulting emulsion showed stable micrometric oil droplets (d50 ≈ 2.8–2.9 μm) and maintained physico-chemical stability, pH, and preserved antioxidant activity over six months. Crucially, a single-blind, placebo-controlled clinical study confirmed the safety (minimal irritation) and efficacy of the CSE-loaded formulation, showing improvements in hydration, elasticity, and reduced wrinkle appearance after just 15 days of daily application (Figure 2) [35]. High volunteer satisfaction underscored the commercial viability of these extracts as safe, effective, and consumer-acceptable natural antioxidants. Moreover, the innovative antimicrobial activity of the aqueous chestnut spiny bur extract would open the doors for novel functional applications in the field of antimicrobial resistance [33].

### 3.5. Limitations

Despite the substantial evidence supporting the chemical richness, safety, and bioefficacy of *C. sativa* spiny bur extracts (Table 2), their transition to large-scale industrial valorization faces several limitations, primarily revolving around standardization. The core challenge lies in the high variability of the bioactive compound content, which is significantly influenced by differences in geographic origin, harvest time, and the specific extraction methodology employed. As noted in reviews of chestnut by-products, these factors variably impact secondary metabolite profiles, making the reproducibility of the final ingredient difficult to guarantee without strict quality control [5]. Furthermore, extraction efficiency is demonstrably influenced by solvent choice and operational conditions, necessitating optimization strategies, such as response surface methodology, to maximize antioxidant recovery while minimizing variability [37].

Process optimization must also address formulation challenges, specifically the impact of high extract concentrations on sensory characteristics. For instance, higher concentrations of spiny bur extracts can impart a dark brown color and intense odor to formulations, potentially reducing consumer acceptance [33]. Additionally, the stability of bioactive compounds is a critical concern; the physical stability of semi-solid formulations can be compromised by the chemical instability of added natural constituents, such as vitamins or polyphenols, which may degrade under certain storage conditions [8,38].

## 4. Chestnut Leaves

### 4.1. Sampling and Extraction Methodologies

The investigation into the phytochemical profile of *C. sativa* leaves has involved a comprehensive sampling of diverse cultivars across a wide latitudinal range in Europe. This approach underscores the research interest in defining a consistent phytochemical core despite environmental variables. Leaf samples include the ‘*Marrone di Roccadaspide*’ PGI cultivar collected in Salerno, Southern Italy [11]; the ‘Longal’ variety sourced from Vinhais, Northeast Portugal [3]; mixed ‘*Castagna*’ and ‘*Marrone*’ cultivars from Granaglione, Bologna, Italy [31]; and the ‘Venegon’ and ‘Verdesa’ varieties from the “Campo dei Fiori” regional park in Varese, Northern Italy [31,39,40]. The harvested material was consistently dried and milled into a fine powder to ensure homogeneity for subsequent analysis.

The complexity of the leaf matrix has necessitated the use of diverse and analytically robust extraction techniques, ranging from traditional maceration to innovative high-energy methods, selected based on the target metabolites. The traditional extraction protocol involved extracting dried leaves sequentially at room temperature with solvents of increasing polarity—petroleum ether, chloroform, and finally, methanol (MeOH)—yielding a crude MeOH extract [11]. Standard maceration was also employed using 80% ethanol (MAC-HE) under continuous stirring [3], or a MeOH/H_2_O (1:1) mixture followed by sonication and centrifugation to obtain a crude leaf extract [31]. For the specific recovery of polyphenols intended for gastric and dermatological applications, simple hydroalcoholic maceration (50:50 ethanol/water) at room temperature for 4 to 16 h was preferred [39,40]. To foster sustainability and efficiency, innovative methods were compared using the ‘Longal’ cultivar as seen for the spiny burs [3]. These included Ultrasound-Assisted Extraction (UAE), performed with 80% ethanol (UAE-HE) or ultrapure water (UAE-W) utilizing a 25 kHz ultrasonic system, and Microwave-Assisted Extraction (MAE), conducted using ultrapure water (MAE-W) in a closed vessel at 80 °C. Additionally, Ultrasound-Assisted Maceration (UAM) with methanol was utilized to maximize yield for rumen fermentation studies [13]. To isolate specific bioactive classes or pure compounds, crude extracts were subjected to sophisticated fractionation workflows [41].

### 4.2. Chemical Composition and Variability

The phytochemical composition of *C. sativa* leaves has been extensively elucidated through progressively sophisticated metabolomic approaches, revealing a remarkably rich and structurally diverse array of polyphenols and specialized metabolites that underpin their emerging bioactive potential. Early work established that chestnut leaves constitute a sustainable polyphenol-rich by-product, containing substantial amounts of hydrolyzable tannins (6–8% *w*/*w* in polar extracts) and total polyphenols exceeding 25% *w*/*w* of the extract (Folin–Ciocalteu assay), including the ellagitannin isomers castalagin and vescalagin previously characterized in bark [42]. Building on this foundation, high-resolution LC–ESI/LTQ-Orbitrap/MS/MS and LC–ESI/QTrap/MS/MS platforms enabled a more refined mapping of leaf chemical diversity, documenting five major metabolite classes—hydrolyzable tannins, flavonoids, triterpenoids, phenol glucoside derivatives, and ellagic acid derivatives [11]. This workflow allowed the annotation of multiple galloyl glucose derivatives and a broad range of galloyl- and HHDP-glucose conjugates, confirming the chemical complexity of tannins in the leaf metabolome [11].

Complementary LC-HRMS profiling further revealed that leaves harbor the richest phenolic repertoire among *C. sativa* by-products, with 22 leaf-specific compounds identified [3]. Leaves shared most phenolics with spiny burs (14/19) while also containing exclusive or abundant molecules such as methylated and glycosylated ellagic acid derivatives, consistent with earlier reports [43,44]. Extensive gallotannins, including cretanin, chesnatin isomers, and chestanin, were also confirmed, alongside a structurally diverse suite of ellagitannins such as bis-HHDP-glucose, galloyl-bis-HHDP-glucose, digalloyl-HHDP-glucose, trigalloyl-HHDP-glucose, and galloyl-gallagyl-hexoside [45,46,47]. Flavonoids represented a second major chemical class, dominated by quercetin, isorhamnetin, and kaempferol glycosides [3]. A deeper structural interpretation of leaf constituents has been achieved through NMR-guided fractionation approaches. Marrazzo et al. (2023) employed solvent partitioning of crude methanolic extracts into hexane, ethyl acetate, butanol, and aqueous fractions, followed by an integrated NMR–UHPLC-MS workflow, demonstrating that solvent polarity drives a clear chemical stratification of fatty acids, flavonoids, sugars, tannins, and organic acids [31]. Hexane fractions were enriched in unsaturated fatty acids, ethyl acetate fractions concentrated flavonol glycosides such as astragalin, isorhamnetin glucoside, and myricitrin, and further purification led to the structural elucidation of a previously undescribed acylated flavonoid—kaempferol 3-rhamnosyl(1 → 6)(2″-*trans*-*p*-coumaroyl)hexoside [31]. Butanol fractions retained lower flavonol concentrations along with tannin-like signals, while the aqueous fraction was dominated by primary metabolites including sucrose, glucose, and quinic acid [31]. These fractionation studies reinforce earlier LC-MS findings showing that chestnut leaves harbor abundant flavonol glycosides and hydrolyzable tannins, including astragalin, quercetin glycosides, chestanin, chesnatin, HHDP derivatives, castalagin, and vescalagin. Further targeted analyses have deepened understanding of ellagitannin distribution and quantification. Piazza et al. confirmed castalagin and vescalagin in hydroalcoholic leaf extracts prepared from two *C. sativa* varieties (var. venegon and var. verdesa) using LC–MS/MS [40]. Extraction yields ranged from 16.24% to 21.88% (dry extract/dry plant material), consistent with earlier reports describing the leaves as a significant natural reservoir of tannins and flavonoids. These combined multi-platform analytical efforts converge to define *C. sativa* leaves as one of the phytochemically richest components of the chestnut tree.

### 4.3. Bioactivity

*C. sativa* leaves represent a biologically rich by-product whose phytochemical composition underpins a broad spectrum of antioxidant, anti-inflammatory, antimicrobial, dermatological, and gastroprotective properties [48]. Their antioxidant capacity has been robustly demonstrated through spectrophotometric assays, where methanolic extracts exhibited strong radical-scavenging ability and notable ferric-reducing power [11]. These findings were reinforced at the cellular level in macrophages, where non-cytotoxic concentrations significantly reduced intracellular ROS, approaching the efficacy of reference standards like quercetin [11]. Comparable antioxidant assays applied to microwave-assisted extracts demonstrated that leaves maintain relevant activity despite variable phenolic content, confirming that efficacy depends on the qualitative profile of phytochemicals [3].

**Figure 3:**
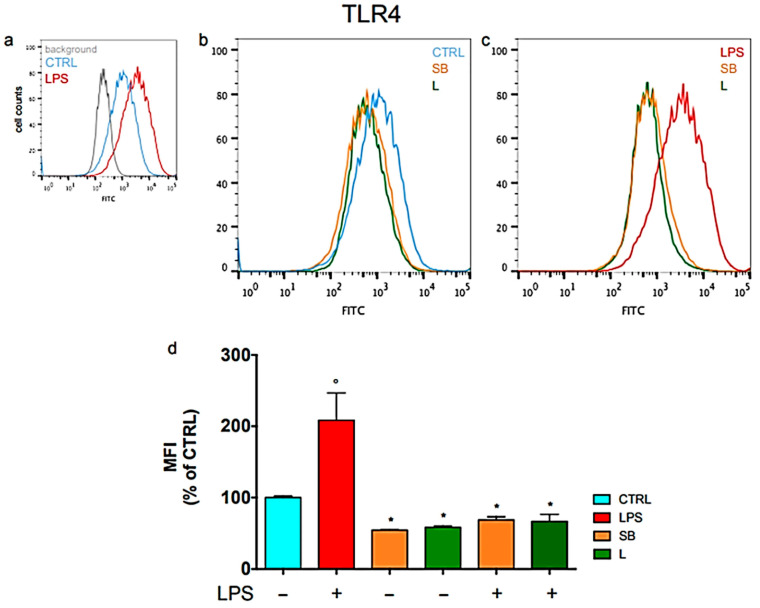
Modulation of Toll-Like Receptor 4 (TLR4) surface expression in BV-2 microglial cells by chestnut extracts. Flow cytometric analysis shows that treatment with Spiny Bur (SB) and Leaf (L) extracts significantly reduces TLR4 levels on the cell surface compared to LPS-stimulated controls, suggesting a protective mechanism against neuroinflammation by limiting receptor availability. (**a**) Representative plots of untreated BV-2 cells (**b**) Representative plots of BV-2 cells not activated with LPS (**c**) Representative plots of LPS-stimulated BV-2 cells (**d**) Relative quantification is expressed as MFI, median fluorescence intensity. Results are expressed as means ± SEM of three independent experiments. Statistical analysis was performed by Fisher’s LSD test following one-way ANOVA. ° *p* < 0.05 significantly different from control cells; * *p* < 0.05 significantly different from LPS-treated cells. Adapted from Marrazzo et al. (2023) [31].

Antioxidant activity closely parallels the leaves’ anti-inflammatory properties. In macrophage models, leaf extracts significantly reduced NF-κB activation and nitrite production, effects likely driven by both ellagitannins and bioactive polar lipids such as glycolipids and lysophospholipids [11,49]. Anti-inflammatory and neuroprotective actions were further validated in microglial cells, where leaf extracts reduced TLR4 membrane expression and suppressed key inflammatory mediators, including iNOS and TNF-α [31] (Figure 3).

The dermatological potential of leaf extracts has been specifically highlighted in the context of acne vulgaris management. In co-culture models mimicking acne inflammation, leaf extracts and purified castalagin exerted a dose-dependent inhibition of pro-inflammatory cytokines IL-8 and IL-6 while maintaining cell viability [39]. Mechanistically, this activity was linked to the suppression of the NF-κB pathway and a modest reduction in AP-1 activation [39]. Crucially, the treatment counteracted the abnormal expression of cytokeratin-10 (CK-10), suggesting a capacity to modulate the hyperkeratinization typical of acne lesions, and significantly inhibited bacterial biofilm formation without affecting the growth of *Cutibacterium acnes*, thereby preserving the skin microbiome [39].

This non-biocidal approach extends to other pathogens. Leaf extracts and specific triterpenoids like Castaneroxy A act as quorum sensing inhibitors against methicillin-resistant *S. aureus* (MRSA) [50]. By blocking toxin production and disrupting the *agr* gene regulator system, these compounds reduce bacterial virulence and dermonecrosis in vivo without exerting the evolutionary pressure that drives drug resistance [50]. Finally, leaf ellagitannins exhibit gastroprotective effects, reducing *H. pylori*-induced inflammation in gastric epithelial cells through the modulation of NF-κB signaling and cytokine release [40,51,52]. Recent studies such as those by Piazza et al. 2023 [40] have addressed the gap in non-biocidal management of gastric inflammation, demonstrating that leaf ellagitannins reduce cytokine release without killing the bacteria.

In the context of acne vulgaris, leaf extracts and purified castalagin inhibited pro-inflammatory cytokine release dose-dependently while maintaining cell viability [39]. Furthermore, the treatment counteracted abnormal keratinocyte differentiation and significantly inhibited bacterial biofilm formation [39]. Finally, leaf ellagitannins exhibit gastroprotective effects; leaf extracts reduced cytokine release in gastric epithelial cells challenged with *H. pylori*, while purified ellagitannins showed even stronger potency [40].

### 4.4. Potential Biotechnological Applications

The diverse bioactivities of *C. sativa* leaf extracts position them as high-value candidates for multiple industrial sectors, aligning with circular economy principles by valorizing agro-forestry waste. In the cosmeceutical and dermatological fields, the leaf extract and its signature ellagitannins offer a novel approach to managing acne vulgaris. Unlike conventional antibiotics that disrupt the skin microbiome, chestnut leaf extracts inhibit *Cutibacterium acnes* biofilm formation and inflammation without affecting bacterial growth, thereby preserving the skin’s ecological balance. Additionally, their ability to modulate keratinization via CK-10 expression suggests potential in treating hyperkeratotic disorders [39].

In the nutraceutical and pharmaceutical sectors, the extracts show promise for managing gastritis. The demonstrated synergy between *C. sativa* and *Cistus incanus* extracts in inhibiting *H. pylori* adhesion and IL-8 release support the development of combined botanical food supplements that are effective at physiologically achievable concentrations [52]. Furthermore, the isolation of castaneroxy A opens avenues for anti-virulence therapies against antibiotic-resistant pathogens like MRSA; by targeting quorum sensing rather than cell viability, this compound reduces selective pressure for resistance, offering a sustainable alternative to traditional antibiotics [50]. Finally, in veterinary applications, leaf extract fractions were evaluated as natural rumen modifiers. Both polar (tannin-rich) and non-polar (lipid/terpenoid-rich) fractions promoted an increase in total volatile fatty acids (VFAs) while decreasing the acetate/propionate ratio. Crucially, the extracts significantly reduced methane (CH4) production, a key environmental target in livestock management [12].

### 4.5. Limitations

Despite the promising applications, several challenges must be addressed to facilitate the industrial translation (Table 3) of *C. sativa* leaf extracts, particularly those rich in ellagitannins and flavonoids. A primary limitation is the chemical variability inherent to botanical sources [11]. Metabolite profiles are significantly influenced by cultivar (e.g., ‘Verdole’ vs. other varieties), pedoclimatic conditions, and the specific extraction methods employed [16]. This necessitates rigorous standardization of the raw material and the development of quality control methods to ensure batch-to-batch consistency for commercial products.

Stability and bioavailability also present hurdles, particularly for oral formulations. In the gastrointestinal tract, ellagitannins like castalagin and vescalagin undergo degradation, which can limit the number of active molecules reaching the target tissues or the bloodstream [40]. While castalagin shows partial resistance to gastric digestion, other bioactive components like vescalagin are more prone to degradation, potentially reducing efficacy in vivo [24]. Furthermore, the anti-inflammatory activity of the extracts, such as that demonstrated against *H. pylori* and *C. acnes*, relies on the stable delivery of these compounds [39,40]. From a safety and toxicological standpoint, while the extracts are generally safe, high concentrations of specific isolated compounds, such as the triterpenoid castaneroxy A, have shown cytotoxicity against human keratinocytes at certain high doses, an observation that must be factored into future cosmetic and topical formulation strategies [39,50]. Additionally, for potential applications in animal feed, high tannin intake in ruminants can negatively impact feed palatability and digestibility, which must be managed through appropriate formulation [12].

## 5. Discussion

The integrated analysis of *C. sativa* by-products supports the concept that shells, spiny burs, and leaves form a complementary “bioactive triad”, rather than interchangeable sources of polyphenols [10,24,31]. Although these matrices share a common phytochemical backbone dominated by hydrolysable tannins and flavonoids, their distinct chemical fingerprints translate into markedly different biological properties and valorization pathways [3]. This functional diversification is a key strength of chestnut residues within a life-science and circular bioeconomy framework (Table 4).

Among the three matrices, spiny burs consistently emerge as the most phenolic-dense fraction, with total phenolic contents reported to reach up to 580 mg GAE/g [10,11]. Their exceptional enrichment in ellagitannins, particularly castalagin and vescalagin, underpins their strong antioxidant and antimicrobial potential [11]. Beyond classical antioxidant activity, in vitro studies indicate that spiny burs can potentiate conventional antibiotics against resistant pathogens such as *H. pylori* and *S. aureus*, positioning them as potential candidates for adjuvant therapies [3,53].

In contrast, chestnut leaves appear to occupy a more specialized biological niche. Rather than acting primarily as biocidal agents, leaf-derived compounds have demonstrated anti-virulence and host-modulatory effects in experimental models. Notably, molecules such as Castaneroxy A have been shown to interfere with bacterial quorum sensing systems, reducing pathogenicity without directly inhibiting bacterial growth [39,50]. This non-biocidal mechanism suggests a theoretical advantage for dermatological applications, such as acne, where preservation of the skin microbiome is critical [39]. However, while reducing selective pressure for resistance offers a conceptual benefit over traditional antimicrobials [42,50], the clinical translation of these anti-virulence effects remains a key challenge that must be addressed in future research.

Chestnut shells, although generally characterized by lower total phenolic content compared to spiny burs and leaves, display distinct strengths from a translational perspective [9,25]. In vivo studies demonstrate that shell extracts can modulate glucose and lipid metabolism in murine models, highlighting their potential role in metabolic health applications [25]. Importantly, their biological activity is supported by evidence of bioaccessibility and intestinal permeability of shell-derived phenolic metabolites, particularly low-molecular-weight phenolic acids capable of crossing the epithelial barrier and exerting systemic hypoglycemic and hypolipidemic effects [20,21,28]. These findings emphasize that bioavailability and metabolic fate, rather than phenolic concentration alone, are critical determinants of functional efficacy.

Despite these promising properties, a major challenge common to all three by-products is the lack of standardization [5,34]. The literature consistently shows that geographic origin, cultivar, and extraction methodology, particularly the choice between conventional and green technologies, substantially influence chemical composition and bioactivity [3,16,18]. For example, high-temperature subcritical water extraction can enhance antioxidant capacity but simultaneously degrade complex tannins into simpler phenols such as pyrogallol, yielding extracts that are not bio-equivalent to those obtained under milder ethanolic conditions [17,18]. This variability complicates reproducibility, comparison across studies, and the development of standardized ingredients suitable for regulatory approval.

Safety considerations further underscore the need for rigorous quality control. Chestnut shells can bioaccumulate potentially toxic elements, including As, Pb, and Hg, particularly in trees grown in mining-impacted areas, necessitating careful sourcing and systematic metal screening [29]. In parallel, the cytotoxicity observed at high doses of certain leaf-derived triterpenoids highlights the importance of dose optimization and toxicological evaluation [39]. Moreover, the strong protein-binding capacity of tannins raises concerns regarding potential interactions with nutrients or pharmaceuticals, indicating that further pharmacokinetic and interaction studies are warranted [9].

From an application standpoint, consumer acceptance and formulation performance remain critical determinants of successful industrial translation. In cosmetic applications, the balance between bioefficacy and sensory attributes such as color, odor, and texture must be carefully optimized, making consumer profiling and formulation studies essential [54]. At present, much of the available evidence is derived from in vitro and ex vivo models, including human gastric epithelial cells infected with *H. pylori* and keratinocytes challenged with *C. acnes* [39,40]. While these models provide valuable mechanistic insights, they cannot fully capture the complexity of human physiology. Consequently, well-designed in vivo studies and clinical trials are required to validate efficacy, determine optimal dosing, and confirm tissue-specific bioavailability of active compounds.

Future research should also prioritize advanced formulation strategies, such as hydrogel systems and nano-encapsulation, to improve stability, delivery, and bioavailability of chestnut-derived bioactives, thereby bridging the gap between laboratory research and commercial products [33,40]. In parallel, expanding the phytochemical knowledge base through comprehensive reviews [55], comparative analyses of antimicrobial tannins [56], and studies on related chestnut tissues such as bark [56] will further refine raw material selection. Addressing bioaccessibility challenges (common to many tannin-rich plant species [57]) will ultimately support the bio-guided development of targeted, safe, and effective formulations [57].

## 6. Conclusions

*C. sativa* by-products (shells, spiny burs, and leaves) are no longer merely agricultural waste but are confirmed as valuable, multifunctional matrices capable of driving a circular bioeconomy. Their valorization supports the development of innovative functional ingredients, from metabolic regulators to non-biocidal anti-infectives, powered by green extraction technologies that align with sustainability goals. However, successful industrial exploitation relies on overcoming the current limitations of chemical variability and safety. Only through the establishment of harmonized extraction protocols, rigorous heavy metal screening, and validation via well-designed clinical trials can these by-products evolve into reliable, standardized, and safe functional ingredients for the global market.

## Figures and Tables

**Table 1:** Comprehensive summary of C. sativa shells, detailing the efficacy of conventional versus green extraction technologies (SWE, SFE, DES), the specific profile of phenolic acids and tannins, and the documented bioactivities ranging from antioxidant, antinflammatory, metabolic, to neuroprotective and antimicrobial.

Extraction Technologies	Chemical Composition	Bioactivity	References
Conventional Extraction (Ethanol/Hydroethanol)	Phenolic Acids: Gallic acid, Ellagic acid	Antioxidant: High reducing power and radical scavenging	[16]
SWE (Subcritical Water Extraction)	Flavonoids: Catechin, Epicatechin, Rutin	Anti-inflammatory: Inhibits NF-κB and cytokines (IL-8, MCP-1)	[17,24]
SFE (Supercritical Fluid Extraction)	Tannins: Condensed tannins and Ellagitannins	Metabolic: Hypoglycemic (α-amylase inhibition) and Hypolipidemic	[20,21]
DES (Deep Eutectic Solvents)	Pigments: Stable natural colorants	Neuroprotective: Acetylcholinesterase inhibition	[22]
UAE/MAE(Ultrasound/Microwave)	Phenolics: High total phenolic content	Antimicrobial: Moderate activity against *S. aureus*, *E. coli*	[3,17]

**Table 2:** Overview of *C. sativa* spiny burs, highlighting extraction methods including cryogenic pre-treatments, the dominance of hydrolysable tannins (castalagin/vescalagin) in the chemical profile, and key biological effects such as antioxidant, antimicrobial, antinflammatory and cosmetic applications.

Extraction Technology	Chemical Composition Recovered	Associated Bioactivity	References
Conventional Maceration (Methanol or Ethanol/Water)	Hydrolysable tannins (castalagin, vescalagin); Phenolic acids	Antioxidant: Superior efficacy (highest TPC)	[10,11]
UAE (Ultrasound-Assisted Extraction)	Ellagic acid, Brevifolin carboxylic acid	Antimicrobial: Effective against Gram-positive strains; Anti-inflammatory	[3,32]
MAE (Microwave-Assisted Extraction)	Tannins and Flavonoids	Antioxidant: High reducing capacity	[3]
Cryogenic Grinding	Intact Polyphenols	Cytoprotective: Indirect enhancement of bioactivity	[24]
Lipophilic Extractions	Polar lipids: Phospholipids, Glycolipids	Anti-inflammatory: Synergistic suppression of TLR4 signaling	[11,31]

**Table 3:** Detailed characterization of *C. sativa* leaves, summarizing extraction strategies for recovering polar versus non-polar fractions, the unique presence of anti-virulence triterpenoids like Castaneroxy A, and specific applications in antivirulence, dermatology, gastroprotection, and veterinary science.

Extraction Technology	Chemical Composition	Bioactivity	References
Conventional Maceration (Methanol/Hydroethanol)	Flavonoids: Quercetin, Isorhamnetin glycosides; Tannins	Antioxidant: Strong radical scavenging; Neuroprotective	[11,31]
UAE (Ultrasound-Assisted Extraction)	Polyphenols: High recovery of bioactive fractions	Antimicrobial: Bacteriostatic activity	[3]
MAE (Microwave-Assisted Extraction)	Phenolics: Comparable yield to conventional methods	Antioxidant: Significant intracellular ROS reduction	[3]
Fractionation (Enriched Fractions)	Triterpenoids: Castaneroxy A; Ellagitannins: Castalagin	Anti-virulence: Quorum sensing inhibition (MRSA); Anti-Acne	[39,50]
Aqueous Extraction	Ellagitannins	Gastroprotective: Inhibition of *H. pylori* adhesion	[40,52]

**Table 4:** Comparative chemical composition of *C. sativa* shells, spiny burs, and leaves, highlighting phenolic abundance and specific bioactive classes.

Sample Matrix	Total Phenolic Content (mg GAE/g)	Total Flavonoid Content	Main Chemical Classes	Other Reported Constituents	Ref.
Shells	315–497 mg GAE/g (SWE)	330–503 mg CE/g	Phenolic acids (Gallic, Ellagic), Condensed tannins	Sugars: ~36%; Lignin, Vitamin E, Amino acids	[10,18,22]
Spiny Burs	~580.44 mg GAE/g (Methanolic) 243.98 mg GAE/g (Aqueous UAE)	87.19 mg rutin/g (Methanolic) 27.54 mg QE/g (Aqueous)	Hydrolysable tannins (Castalagin, Vescalagin)	Polar lipids: Phospholipids, Glycolipids, Sphingolipids	[11,24,32]
Leaves	298.96 mg GAE/g (Methanolic)	45.54 mg rutin/g	Hydrolysable tannins, Glycosylated flavonoids	Triterpenoids: Castaneroxy A; Polar lipids	[11,31,42]

## Data Availability

No new data were created.
